# Cancer registries and data protection in the age of health digital interoperability in Europe: The perspective of the Italian Network of Cancer Registries (AIRTUM)

**DOI:** 10.3389/fonc.2022.1052057

**Published:** 2022-12-06

**Authors:** Walter Mazzucco, Fabrizio Stracci, Gemma Gatta, Angelo D’Argenzio, Ettore Bidoli, Simona Carone, Susanna Vitarelli, Maurizio Castelli, Santo Fruscione, Francesco Vitale

**Affiliations:** ^1^ Department of Oncology and Public Health, Executive Board of the Italian Network of Cancer Registries (AIRTUM), Milan, Italy; ^2^ Clinical Epidemiology Unit and Palermo Province Cancer Registry, University Hospital “P. Giaccone”, Palermo, Italy; ^3^ College of Medicine, University of Cincinnati, Cincinnati, OH, United States; ^4^ Umbria Regional Cancer Registry, Department of Medicine and Surgery, University of Perugia, Perugia, Italy; ^5^ Research Department, IRCCS Foundation National Institute Tumor, Milan, Italy; ^6^ Regional Epidemiological Observatory - Campania Region, Naples, Italy; ^7^ Friuli-Venezia-Giulia Regional Cancer Registry, IRCCS Oncological Reference Centre of Aviano (CRO), Aviano, Italy; ^8^ Apulia Regional Cancer Registry, Local health agency of Taranto, Taranto, Italy; ^9^ Marche Regional Cancer Registry, University of Camerino, Camerino, Italy; ^10^ Valle d’Aosta Cancer Registry, Local health agency of Valle d’Aosta, Aosta, Italy

**Keywords:** cancer registries, cancer epidemiology, data protection, digitalization, interoperability

## Introduction

Population-based cancer registries (PBCRs) are advanced public health systems providing ongoing surveillance through systematic collection, analysis, interpretation, and dissemination of high-quality data on cancer incident cases registered in a defined population residing in a specific geographical area (1, 2). Basically, PBCRs are well equipped for strengthening cancer surveillance, playing a strategic role in making geographic and temporal variation comparisons to highlight cancer epidemics, while assessing the effectiveness of preventive interventions and oncological care (3). Furthermore, many PBCRs provide cancer risk communication to local communities and authorities by using valuable tools to spread epidemiological data on cancer in intelligible ways to better address preventive intervention and changes in lifestyles (4). To these ends, PBCRs link records and merge data from different administrative, demographic and health sources, following international standards, recommendations, and guidelines (5–7). Standards in cancer surveillance have been defined in Europe and several cancer research domains are continuously alimented by the cancer registries networks in support of public health and clinical research, with specific regard to aetiologic research, mass screening evaluation, quality of care, translational prognostics, and survivorship (3). More recently, progress has been documented regarding the PBCRs’ capability to estimate the effectiveness of immunisation programmes against vaccine preventable viral infections associated to cancer as well as of cancer screening programmes to decrease late-stage incidence and mortality (8–10). Much more fruits can be harvested from “RegisTrees” having strong roots (11), including the ones allowing the deterministic linkage between cancer registries and clinical registries to generate real-world evidence on treatments, particularly on elderly patients as well, a target population that is usually not included in clinical trials (12, 13).

However, the latest revision of the European Union (EU) data protection framework and of the General Data Protection Regulation (GDPR), while suggesting a harmonization of health registries requirements for confidentiality and individual consent to data processing, made it raise concerns from researchers and epidemiologists experiencing some excessive restrictions that may hamper data linkages between cancer registries and other sources (14–16).

As documented by a survey on how GDPR could have impacted the running of the daily activities of the cancer registries in different EU countries, conducted in 2018 by the European network of cancer registries (ENCR), some critical points related to the implementation and interpretation of the GDPR emerged from PBCRs (17, 18).

Of interest, in 2017 the European Commission adopted a strategy to implement an interoperability framework, in order to improve “the ability of organizations to interact towards mutually beneficial goals, involving the sharing of information and knowledge by means of the exchange of data between the respective ICT systems” (19). As the digital interoperability was conceived to maximize the use of huge amounts of data, by contrast an unnecessarily strict interpretation of EU data protection regulation may lead to missed data linkages in cancer registration and other epidemiological activities (20). Therefore, the nature and the use of data from cancer registries in public health and research purposes, and their potentialities in the age of health digital interoperability, are herein discussed (16).

## Cancer registries and data protection

Within the EU framework the definition of anonymous data is stringent (an individual cannot be identified or identifiable) and open data sharing is likely to violate the GDPR when data aren’t truly anonymised and, thus, not covered by the regulatory indications (14). For these reasons, a specific informed consent from participants is required to make pseudonymised individual data open and accessible, including an explanation of the risk of backwards identification and a disclosure explaining to data subjects what their rights are and how to exercise them (21). Therefore, making pseudonymised individual data derived from primary sources openly accessible may represent a potential threat to the privacy of those patients whose data were already included in cancer registries and are accessed on a retrospective perspective, so posing substantial obstacles to the sharing of data to validate the research results. Moreover, beyond the research purposes, whereas PBCRs institutionally share confidential data with higher administrative levels of surveillance (i.e. from provincial to regional or from regional to national level) to support case aggregation across different areas, a strict interpretation of the rules on confidentiality may introduce differences within a country. The same criticisms may arise of data sharing from one country to another. One possible solution, that has been positively experienced by PBCRs, is the publication on the institutional websites of a disclosure informing patients of the retrospective use of their data for research purposes, while assuring confidentiality and security of the individual data management.

However, even when data are fully anonymised to be shared for research purposes, some concerns might arise from local data protection officers (DPOs) because of a theoretical data breach, potentially allowing hackers to match or bridge information that could make an individual identifiable. Anyway, as access to a primary source itself may imply the possible identification of a patient, the right balance between the risk of potential data confidentiality breach, which is usually very low in PBCRs, and benefits of improving knowledge to prevent and treat cancer, should be considered.

## Cancer registries and digital interoperability: Challenges and perspectives in Italy

The recent progress in digitalisation applied to healthcare data flows has been improving the capability of PBCRs to support cancer surveillance, research, and health services planning, because of a reduction in the lag-time needed to produce cancer incidence data (22). These advancements in cancer registration have at the same time projected the PBCRs towards the new horizons of interoperability and federated networks in healthcare (23). If, on one side, development of advanced cancer surveillance systems based on data-driven models, machine learning and artificial intelligence techniques, may be supported, on the other side, legislation implemented to improve access to personal data could reveal to be a double-edged sword, whereas complexity and implications from misinterpretations may sometimes paradoxically result in data becoming more siloed (23). Thus, today more than ever, the design and the establishment of a cancer registry is a complex process requiring updated evidence-based recommendations as well as substantial resources and careful planning (24).

The new frontiers of integrating epidemiological data with data on health determinants and lifestyles profiling, or with information from environmental monitoring, while interoperating different institutional data flows and data sources based on information communication technology, may improve the knowledge on the effects of exposures on health outcomes across a lifespan in different target populations by using suitable data mining solutions (25). Nevertheless, as volume, accuracy and precision of digital geographic information have increased, and the use of sensors and wearable devices may provide more precise data on individual exposures, more concerns regarding individual privacy and confidentiality might come to the forefront (26). This could be particularly relevant when treating data on rare cancer, or other sensitive topics such as childhood tumours, coming from small communities and, therefore, some useful geospatial cryptography secure solutions have been proposed and successfully implemented (26, 27).

Italy documents a very strong tradition in cancer registration (28). PBCRs are part of the public National Health System and, in 2019, a national network of cancer registries was established, opening to active contribution of the stakeholders (e.g. scientific societies, professionals, associations of cancer patients and citizens, etc.) (29, 30). Anyway, as of today, the implementation process of this network aimed at supporting the Italian national cancer registry, whose regulation is in charge of the Ministry of Health in collaboration with the data protection authority, is still ongoing (31). Since the GDPR implementation, professionals (researchers, epidemiologists, registrars) operating in PBCRs have been experimenting on the field a bureaucratization drift in cancer registration, and among the Italian Network of Cancer Registries (AIRTUM) a debate on how to avert or prevent this phenomenon has arisen (32). Both a bureaucratic attitude (eg. denying of data access to provincial CRs because the transmission procedure in place was conceived for regional CRs only) and a defensive approach (eg. retrospectively requiring the permission of the patient to access the clinical record) from DPOs to data sharing between health institutions may also have a negative impact on completeness or timeliness of data production, thus limiting the role of cancer registries in supporting public health, epidemiology, and outcomes research.

More recently, within the National Recovery and Resilience Plan (NRRP), funded by the EU to support Italy in facing the effects of the COVID-19 pandemic, the institution of a National Health Prevention Hub has been implemented and, within the national complementary plan to NRRP, funded by the Italian government, health and environment protection agencies have been engaged in the establishment of a national health-environment-climate prevention system (31, 33).

In both these frameworks, whereas many efforts are addressed in the development of advanced digital functions and tools to allow a full interoperability of health and environmental data sources for innovative community-based surveillance and prevention of diseases, PBCRs are expected to play a central role. These are all extraordinary opportunities that can’t be missed, and the Italian experience may represent a model to be exported to other EU member states (34).

## Discussion

In the last decades in Europe, PBCRs acquired extensive experience in generating valid information on cancer epidemiology, assessing effectiveness of preventive intervention and treatment patterns, and supporting health-service management and planning, being involved in a continuous effort to provide an optimal cancer surveillance through intensive collaboration with professional and institutional stakeholders (3, 11, 35, 36). Anyway, intensity of use and data quality of PBCRs have been varying depending on the role they played in the politico, onco-medical and public health settings within each country (37).

In the recent years, according to the GDPR and the technological advancements, several anonymization protocols and procedures have been developed to avoid reidentification across the cancer registration process, including simply categorizing variables into broader categories to add noise to the data (38). In addition, innovative methods have been validated for probabilistic patient-level linkage of health data registries without a unique identifier or on encrypted identifying data, thus preserving integrity and privacy (39, 40). Also, the sharing of good practices for data linkage within EU member states has been proposed (41). Although information technology has played a positive role in quality improvement and facility of cancer registries, more strict restriction strategies, such as identifying authentication levels, controlling and coding data approaches, tailored de-identification methods, and other technical measures, had to be developed to secure the patients’ privacy (22, 42, 43).

More recently, the COVID-19 pandemic emergency has changed the scenario, whereas the public health interest prevailed over the privacy and confidentiality of individuals (44), highlighting the vital importance of digital transformation applied to epidemiological surveillance and contact tracing in offering data-minimizing solutions while protecting fundamental rights (45).

The new interoperability framework implemented by the European Commission was conceived to facilitate the digital transformation and to fill the digital gap and divide, while putting citizens at the core of the system, through the use of digital tools in a trustworthy manner, also for health purposes including cancer prevention (46). Changing the paradigm of interdependencies and collaborations through the introduction of an interoperability culture among stakeholders within the healthcare ecosystems, including cancer registries and other epidemiological surveillance systems and health data sources, has the potential to accelerate the digital transformation to satisfy the citizens’ health demand and to bring a social change at the same time, but clear policies, guidelines and governance at legal, organizational, semantic and technical levels, have to be established on a European level (46). Additionally, challenges of health interoperability may be even more complex with the free movement of European citizens across member states, because cross-border healthcare requires setting up shared practices with respect to patients’ data exchange across the different EU countries (47).

Lastly, activities of relevant public health interest, such as cancer surveillance and research, where individual data are used to improve patients’ outcomes and to preserve population health, should be addressed to avoid any limitation related to privacy.

For all the above-mentioned reasons, researchers and epidemiologists are expected to propose a permanent alliance with policymakers, data protection authorities, and citizens to move over any heterogeneity of application in the privacy rules that may limit the widespread evolution of cancer surveillance and research. Moreover, the scientific societies and the ENCR are called for a joint effort to provide methodological guidelines and recommendations, based on the added value of digitalisation and on a multi-professional approach, to support research in cancer epidemiology while preserving the privacy rights of individuals. Through the use of the recalled innovative digital approaches and models, new frontiers of cancer surveillance can be reached and any privacy threats, including the related ethical, legal, and social issues, could be overcome in the near future.

## Author contributions

All individuals listed as authors have contributed to designing, performing or reporting the study and every specific contribution is indicated as follows. WM, FS, and FV: conception and design of the study. WM and FV: supervision. WM, FS, SF, and FV: manuscript writing and drafting. WM, FS, GG, AD’A, EB, SC, SV, MC, SF, and FV: revision of the manuscript. WM, FS, GG, AD’A, EB, SC, SV, MC, SF, and FV: approval of the final version of the manuscript. All authors contributed to the article and approved the submitted version.

## References

[1] Centers for Disease Control and Prevention . Updated guidelines for evaluating public health surveillance systems: recommendations from the guidelines working group (2001). Available at: https://www.cdc.gov/mmwr/preview/mmwrhtml/rr5013a1.htm (Accessed 13 September 2022).

[2] National Institute of Health . What is a cancer registry? (2022). Available at: https://seer.cancer.gov/registries/cancer_registry/cancer_registry.html (Accessed 13 September 2022).

[3] Coebergh JW van den Hurk C Rosso S Comber H Storm H Zanetti R . EUROCOURSE lessons learned from and for population-based cancer registries in Europe and their programme owners: Improving performance by research programming for public health and clinical evaluation. Eur J Cancer (2015) 51(9):997–1017. doi: 10.1016/j.ejca.2015.02.018 25956208

[4] Mazzucco W Cusimano R Zarcone M Mazzola S Vitale F . Funnel plots and choropleth maps in cancer risk communication: a comparison of tools for disseminating population- based incidence data to stakeholders. BMJ Open (2017) 7:e011502. doi: 10.1136/bmjopen-2016-011502 PMC5387987 28363917

[5] World Health Organization . Standards and guidelines for cancer registration in Europe IARC technical publication no. 40 (2003). Available at: https://publications.iarc.fr/Book-And-Report-Series/Iarc-Technical-Publications/Standards-And-Guidelines-For-Cancer-Registration-In-Europe-2003 (Accessed 13 September 2022).

[6] International Association of cancer registries . Guidelines on confidentiality for population-based cancer registration (2004). Available at: https://gicr.iarc.fr/static/public/docs/Confidentiality%20IACR-IARC%20(Tech%20Rep%202004-3).pdf (Accessed 13 September 2022).

[7] World Health Organization . International classification of diseases for oncology –ThirdClassification (2013). Available at: https://apps.who.int/iris/bitstream/handle/10665/96612/9789241548496_eng.pdf (Accessed 13 September 2022).

[8] Falcaro M Castañon A Ndlela B Checchi M Soldan K Lopez-Bernal J . The effects of the national HPV vaccination programme in England, UK, on cervical cancer and grade 3 cervical intraepithelial neoplasia incidence: a register-based observational study. Lancet (2021) 398(10316):2084–92. doi: 10.1016/S0140-6736(21)02178-4 34741816

[9] Vicentini M Zorzi M Bovo E Mancuso P Zappa M Manneschi G . Colorectal cancer screening IMPATTO study working group. impact of screening programme using the faecal immunochemical test on stage of colorectal cancer: Results from the IMPATTO study. Int J Cancer (2019) 145(1):110–21. doi: 10.1002/ijc.32089 30585621

[10] Zorzi M Mangone L Sassatelli R Baracco S Budroni M Castaing M . Characteristics of the colorectal cancers diagnosed in the early 2000s in italy. figures from the IMPATTO study on colorectal cancer screening. Epidemiol Prev (2015) 39(3 Suppl 1):108–14.

[11] Coebergh JW van den Hurk C Louwman M Comber H Rosso S Zanetti R . EUROCOURSE recipe for cancer surveillance by visible population-based cancer RegisTrees in Europe: From roots to fruits. Eur J Cancer (2015) 51(9):1050–63. doi: 10.1016/j.ejca.2015.02.017 25934439

[12] Franchi M Barni S Tagliabue G Ricci P Mazzucco W Tumino R . Effectiveness of first-line bevacizumab in metastatic colorectal cancer: The observational cohort study GRETA. Oncologist (2019) 24(3):358–65. doi: 10.1634/theoncologist.2017-0314 PMC6519764 30097524

[13] Mazzucco W Rossi M Cusimano R Franchi M Bonifazi M Mistretta A . Use of trastuzumab for breast cancer: the role of age. Curr Pharm Des (2014) 20(38):5957–62. doi: 10.2174/1381612820666140314155406 24641226

[14] Regulation (EU) no 2016/679 of the European parliament and of the council of 27 April 2016 on the protection of individuals with regard to the processing of personal data and on the free movement of such data and repealing directive 95/46/EC (General data protection regulation) (2016). Available at: https://www.garanteprivacy.it/regolamentoue (Accessed 13 September 2022).

[15] Regulation (EU) 2018/1725 regulation (EU) 2018/1725 of the European parliament and of the council of 23 October 2018 on the protection of natural persons with regard to the processing of personal data by the union institutions, bodies, offices and agencies and on the free movement of such data, and repealing regulation (EC) no 45/2001 and decision no 1247/2002/EC (Text with EEA relevance (2018). Available at: https://eur-lex.europa.eu/legal-content/EN/TXT/?uri=celex%3A32018R1725 (Accessed 13 September 2022).

[16] Andersen MR Storm HH Eurocourse Work Package 2 Group . Cancer registration, public health and the reform of the European data protection framework: Abandoning or improving European public health research? Eur J Cancer (2015) 51(9):1028–38. doi: 10.1016/j.ejca.2013.09.005 24120502

[17] The European Network of Cancer Registries (ENCR) . . Available at: https://www.encr.eu (Accessed 13 September 2022).

[18] The European Commission’s science and knowledge service . ENCR questionnaire on the impact of the general data protection regulation on cancer registries' activities . Available at: https://www.encr.eu/sites/default/files/2018-ENCRConference/WS%20Data%20Protection%20Neamtiu.pdf (Accessed 13 September 2022).

[19] Eur-Lex . Communication from the commission to the european parliament, the council, the european economic and social committee and the committee of the regions European interoperability framework (2017). Available at: https://eur-lex.europa.eu/legal-content/EN/TXT/?uri=COM:2017:134:FIN (Accessed 13 September 2022).

[20] Braunstein ML . Healthcare in the age of interoperability: The promise of fast healthcare interoperability resources. IEEE Pulse (2018) 9(6):24–7. doi: 10.1109/MPUL.2018.2869317 30452344

[21] Ursin G Malila N Chang-Claude J Gunter M Kaaks R Kampman E . Sharing data safely while preserving privacy. Lancet (2019) 394(10212):1902. doi: 10.1016/S0140-6736(19)32603-0 31708190

[22] Mohammadzadeh N Safdari R Rahimi A . Positive and negative effects of IT on cancer registries. Asian Pac J Cancer Prev (2013) 14(7):4455–7. doi: 10.7314/apjcp.2013.14.7.4455 23992019

[23] Hallock H Marshall SE 't Hoen PAC Nygård JF Hoorne B Fox C . Federated networks for distributed analysis of health data. Front Public Health (2021) 9:712569. doi: 10.3389/fpubh.2021.712569 34660512 PMC8514765

[24] Wormald JS Oberai T Branford-White H Johnson LJ . Design and establishment of a cancer registry: a literature review. ANZ J Surg (2020) 90(7-8):1277–82. doi: 10.1111/ans.16084 32564454

[25] Bianconi F Brunori V Valigi P La Rosa F Stracci F . Information technology as tools for cancer registry and regional cancer network integration. IEEE Trans Systems Man Cybernetics - Part A: Syst Humans (2012) 42(6):1410–24. doi: 10.1109/TSMCA.2012.2210209

[26] Jacquez GM Essex A Curtis A Kohler B Sherman R Emam KE . Geospatial cryptography: enabling researchers to access private, spatially referenced, human subjects data for cancer control and prevention. J Geogr Syst (2017) 19(3):197–220. doi: 10.1007/s10109-017-0252-3 29085255 PMC5659297

[27] Mazzucco W Cusimano R Mazzola S Rudisi G Zarcone M Marotta C . Childhood and adolescence cancers in the Palermo province (Southern italy): Ten years (2003-2012) of epidemiological surveillance. Int J Environ Res Public Health (2018) 15(7):1344. doi: 10.3390/ijerph15071344 29949937 PMC6069060

[28] Guzzinati S Spitale A . The Italian network of cancer registries (AIRT). Epidemiol Prev (2004) 28(2 Suppl):7–11. https://pubmed.ncbi.nlm.nih.gov/15281599/ 15281599

[29] Italian Parliament decree-law n. 179 (2012). (GU n.245 del 19-10-2012 - Suppl. Ordinario n. 194).

[30] Italian Parliament law n. 29 (2019). (GU n.81 del 05- 04-2019).

[31] Italian Government . NRPP, mission 6 "Health" and specifically the realization of the national health prevention hub (M6C2, investment 1.3.2) (2021). Available at: https://www.mef.gov.it/en/focus/documents/PNRR-NEXT-GENERATION-ITALIA_ENG_09022021.pdf (Accessed 13 September 2022).

[32] Associazione Italiana Registri Tumori AIRTum . Corso di aggiornamento per operatori dei registri tumori . Available at: https://www.registri-tumori.it/cms/eventi/corso-di-aggiornamento-operatori-dei-registri-tumori (Accessed 13 September 2022).

[33] Art. 27, decree law n. 36 (2022). Available at: https://www.gazzettaufficiale.it/eli/id/2022/06/29/22A03859/sg (Accessed 13 September 2022).

[34] Italian Parliament decree-law n. 59 (2021). Available at: https://www.gazzettaufficiale.it/eli/id/2021/05/07/21G00070/sg (Accessed 13 September 2022).

[35] Greene DN McClintock DS Durant TJS . Interoperability: COVID-19 as an impetus for change. Clin Chem (2021) 67(4):592–5. doi: 10.1093/clinchem/hvab006 PMC7929044 33475729

[36] Roder D . What roles should population-based cancer registries be playing in the 21st century? reflections on the Asian cancer registry forum, Bangkok, February 2014. Asian Pac J Cancer Prev (2014) 15(5):1895–6. doi: 10.7314/apjcp.2014.15.5.1895 24716907

[37] Siesling S Louwman WJ Kwast A van den Hurk C O'Callaghan M Rosso S . Uses of cancer registries for public health and clinical research in Europe: Results of the European network of cancer registries survey among 161 population-based cancer registries during 2010-2012. Eur J Cancer (2015) 51(9):1039–49. doi: 10.1016/j.ejca.2014.07.016 25131265

[38] Ursin G Sen S Mottu JM Nygård M . Protecting privacy in Large datasets-first we assess the risk; then we fuzzy the data. Cancer Epidemiol Biomarkers Prev (2017) 26(8):1219–24. doi: 10.1158/1055-9965.EPI-17-0172 28754793

[39] Heins MJ de Ligt KM Verloop J Siesling S Korevaar JC . Opportunities and obstacles in linking large health care registries: the primary secondary cancer care registry - breast cancer. BMC Med Res Methodol (2022) 22(1):124. doi: 10.1186/s12874-022-01601-0 35477392 PMC9044735

[40] Bartholomäus S Siegert Y Hense HW Heidinger O . Secure linking of data from population-based cancer registries with healthcare data to evaluate screening programs. Gesundheitswesen (2020) 82(S02):S131–8. doi: 10.1055/a-1031-9526 31822021

[41] Good Practice Data Linkage (GPD) . A translation of the German version. Int J Environ Res Public Health (2020) 17(21):7852. doi: 10.3390/ijerph17217852 33120886 PMC7663300

[42] Gal TS Tucker TC Gangopadhyay A Chen Z . A data recipient centered de-identification method to retain statistical attributes. J BioMed Inform (2014) 50:32–45. doi: 10.1016/j.jbi.2014.01.001 24412834

[43] Yoon HJ Stanley C Christian JB Klasky HB Blanchard AE Durbin EB . Optimal vocabulary selection approaches for privacy-preserving deep NLP model training for information extraction and cancer epidemiology. Cancer biomark (2022) 33(2):185–98. doi: 10.3233/CBM-210306 PMC9377550 35213361

[44] Kaplan B . Revisiting health information technology ethical, legal, and social issues and evaluation: telehealth/telemedicine and COVID-19. Int J Med Inform (2020) 143:104239. doi: 10.1016/j.ijmedinf.2020.104239 33152653 PMC7831568

[45] Abeler J Bäcker M Buermeyer U Zillessen H . COVID-19 contact tracing and data protection can go together. JMIR Mhealth Uhealth (2020) 8(4):e19359. doi: 10.2196/19359 32294052 PMC7173240

[46] Kouroubali A Katehakis DG . The new European interoperability framework as a facilitator of digital transformation for citizen empowerment. J BioMed Inform (2019) 94:103166. doi: 10.1016/j.jbi.2019.103166 30978512

[47] Gavrilov G Vlahu-Gjorgievska E Trajkovik V . Healthcare data warehouse system supporting cross-border interoperability. Health Inf J (2020) 26(2):1321–32. doi: 10.1177/1460458219876793 31581924

